# Immunostimulatory Effect of Sulfated Galactans from the Green Seaweed *Caulerpa cupressoides* var. *flabellata*

**DOI:** 10.3390/md18050234

**Published:** 2020-04-29

**Authors:** Jefferson da Silva Barbosa, Diego Araújo Sabry, Cynthia Haynara Ferreira Silva, Dayanne Lopes Gomes, Arquimedes Paixão Santana-Filho, Guilherme Lanzi Sassaki, Hugo Alexandre Oliveira Rocha

**Affiliations:** 1 Laboratório de Biotecnologia de Polímeros Naturais—BIOPOL, Departamento de Bioquímica, Universidade Federal do Rio Grande do Norte, Natal 59.078-970, Rio Grande do Norte, Brazil; jefferson.barbosa@ifrn.edu.br (J.d.S.B.); popoh.diego@gmail.com (D.A.S.); cynthiahaynara@gmail.com (C.H.F.S.); 2 Programa de Pós-Graduação em Ciências da Saúde, Universidade Federal do Rio Grande do Norte (UFRN), Natal 59012-570, Rio Grande do Norte, Brazil; 3 Instituto Federal de Educação, Ciência e Tecnologia do Rio Grande do Norte (IFRN)—Campus, São Gonçalo do Amarante 59291-727, Rio Grande do Norte, Brazil; 4 Instituto Federal de Educação, Ciência e Tecnologia do Piauí (IFPI)—Campus, BR 020, s/n, São Raimundo Nonato 64770-000, Bairro Primavera, Brazil; dayanne.oliveira@ifpi.edu.br; 5 Departamento de Bioquímica e Biologia Molecular, Universidade Federal do Paraná (UFPR), Curitiba 81.531-980, Paraná, Brazil; arquimetal@gmail.com (A.P.S.-F.); sassaki@ufpr.br (G.L.S.)

**Keywords:** sulfated polysaccharides, galactans, green seaweed, NMR, immunostimulation, inflammatory mediators

## Abstract

Sulfated polysaccharides (SPs) obtained from green seaweeds are structurally heterogeneous molecules with multifunctional bioactivities. In this work, two sulfated and pyruvated galactans were purified from *Caulerpa cupressoides* var. *flabellata* (named SP1 and SP2), and their immunostimulatory effect was evaluated using cultured murine macrophage cells. Both SPs equally increased the production of nitric oxide, reactive oxygen species, and the proinflammatory cytokines TNF-α and IL-6. NMR spectroscopy revealed that both galactans were composed primarily of 3)-β-d-Galp-(1→3) units. Pyruvate groups were also found, forming five-membered cyclic ketals as 4,6-*O*-(1’carboxy)-ethylidene-β-d-Galp residues. Some galactoses are sulfated at C-2. In addition, only SP2 showed some galactose units sulfated at C-4, indicating that sulfation at this position is not essential for the immunomodulatory activity of these galactans. Overall, the data showed that the galactans of *C. cupressoides* exhibited immunostimulating activity with potential therapeutic applications, which can be used in the development of new biomedical products.

## 1. Introduction

Current epidemiological data indicate an increase in immunological diseases. This has stimulated the search for a class of molecules, generally called immunomodulatory molecules, capable of increasing or suppressing the immune response in immune-mediated diseases [1].

Research on natural compounds that can modulate the immune response has become a focus in the experimental field, since such compounds have potential applications in the areas of immunopharmacology and oncotherapy. Different authors have reported that polysaccharides obtained from plants, fungi, and seaweed are able to modify various cellular processes and consequently possess a variety of bioactivities, particularly potent effects on immune function [2,3,4]. Thus, immunostimulatory compounds, such as sulfated polysaccharides (SPs), have potential applications in the treatment of infections, immunodeficiencies, and cancer [5].

In this context, macrophages have been used as a study model in the identification of compounds with immunomodulatory properties [6,7]. Together with neutrophils, these cells constitute the body’s first line of defense [8]. Through their antigen-presenting ability, they also play an important role in adaptive immunity [9]. Activation of macrophages is a key event in innate and adaptive immunity and is essential for defense mechanisms. Once activated by foreign agents, these cells can phagocytose and kill microorganisms and tumor cells, as well as produce molecules that recruit and activate other cells to the site of infection. In response to this stimulus, macrophages increase the production of reactive nitrogen intermediates such as nitric oxide (NO), reactive oxygen species (ROS), and proinflammatory cytokines such as TNF-α and IL-6 [10]. Therefore, macrophages are often used to evaluate the immunomodulatory effects of bioactive compounds from natural sources [11,12].

Although an increasing number of studies on the immunomodulatory activity of SPs from green seaweeds are being conducted, the extent of their activities varies greatly among different species of seaweed, and the structure correlations remain undefined [13].

The SPs of green seaweeds consist mainly of galactose, xylose, arabinose, mannose, rhamnose, glucuronic acid, and/or glucose [14]. The proportion of these monosaccharides seems to differ with the genus of the seaweed; for example, in seaweeds of the genera *Monostroma* and *Ulva*, it is more common to find rhamnose [15], whereas homo- and heterogalactans are common in the seaweeds of the genus *Caulerpa* [16], which are known to synthesize polysaccharides with immunomodulatory activities. Xylogalactans from *Caulerpa lentillifera* were found to exhibit a potent immunomodulatory effect by stimulating phagocytosis and increasing NO and cytokine secretion in RAW 264.7 macrophages [17]. In another work, Sun et al. [18] found that xylogalactomannans increased cell proliferation, phagocytosis, NO secretion, and alkaline phosphatase activity in macrophages. Galactans of *C. cupressoides* var. *lycopodium* showed antinociceptive and anti-inflammatory effects in vivo by reducing leukocyte migration in the peritoneal cavity of rats [19], while galactans of *Caulerpa mexicana* showed antinociceptive effect and decreased paw edema and myeloperoxidase activity in mice [20]. Ribeiro et al. [21] observed that *Caulerpa racemosa* SPs also exhibited antinociceptive and anti-inflammatory activities in an in vivo mouse model.

Less information is available on the SPs of the macroalga *C. cupressoides* var. *flabellata* and their immunomodulatory effects. This species synthesizes SPs that have antioxidant, anticoagulant, and antiproliferative activities in vitro [22]. Costa et al. [23] obtained four SPs populations with different characteristics regarding molecular weight and sulfate content. Monosaccharide composition analysis showed that galactose was the main constituent for all fractions; although glucose, mannose, xylose, rhamnose, and fucose were found, their proportions were different in each fraction. Subsequently, Barbosa et al. [24] investigated the immunostimulatory potential of these four polysaccharide fractions to determine their capacity to stimulate the production of different inflammatory mediators in the RAW 264.7 cell line. Of these, the so-called CCB-F1.0 fraction had a potent effect on the production of NO, ROS, and the cytokines TNF-α and IL-6. Although the SPs of the CCB-F1.0 fraction were determined to be composed of 76.47% total sugars and 17.95% sulfate, have a molecular weight of 155 kDa, and consist of galactose, mannose, and xylose in the ratio of 1.0:0.1:0.6 [23], further structural details of the polysaccharides of this fraction were not determined. Therefore, the present work aimed to purify the SPs of the CCB-F1.0 fraction of *C. cupressoides* var. *flabellata* and to characterize them structurally using nuclear magnetic resonance spectroscopy. In addition, to confirm that CCB-F1.0 immunomodulatory activity came from these molecules, the effects of SPs on some immunostimulating mediators (NO, ROS, and cytokines) using RAW 264.7 murine macrophages cells were investigated.

## 2. Results and Discussion

### 2.1. Purification of SPs

**Figure 1:**
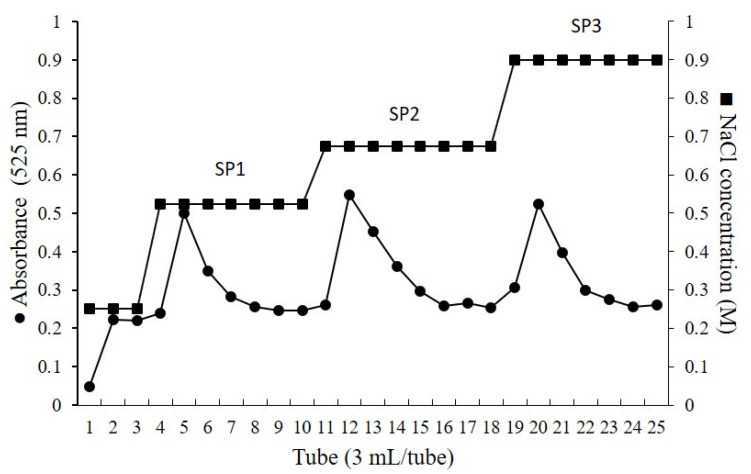
Stepwise elution profile of SPs from *C. cupressoides* on a HiTrap DEAE FF column.

Initially, to obtain the SPs-rich extracts, proteolysis and methanol precipitation steps were performed. After these procedures, the SPs were fractioned with increasing volumes of propanone until the fraction CCB-F1.0 was obtained as described by Costa et al. [23]. Then, the SPs of this fraction were purified by ion exchange chromatography and eluted in a stepwise NaCl gradient (0.25–1.0 M). As shown in Figure 1, the elution of CCB-F1.0 (50 mg) generated three peaks in the chromatogram, corresponding to the molarities of 0.525 M (15.5 mg), 0.675 M (4.2 mg), and 0.9 M (1.8 mg), respectively. Based on this elution profile, the SPs obtained were labeled SP1, SP2, and SP3. Furthermore, SP1, SP2, and SP3 yielded 72%; 19.5%, and 8.5% (w/w) of the eluted SPs present in CCB-F1.0, respectively. Because of the lower yield obtained in the SP3 purification, the following analyses were not performed with this material. 

Physicochemical analysis showed that SP1 and SP2 were composed mainly of polysaccharides and low quantity of protein, ~0.25% and 0.20%, respectively. In addition, both SPs were composed mainly of galactose, sulfate, and traces of mannose, as seen in Table 1.

SP1 and SP2 were further analyzed by gel permeation chromatography (GPC) in a Sephadex^®^ G-100 column to determine their homogeneity, as seen in Figure S1. The chromatograms of the *Caulerpa* SPs showed a single peak. Furthermore, the chromatogram obtained from GPC was used to calculate the apparent molecular weight using a regression equation determined using different molecular weight standards. Thus, the molecular weight of SP1 and SP2 was found to be 125 and 135 kDa, respectively. These values were similar to those demonstrated by Costa el al. [23] in the studies of SPs in the CCB-F1.0 fraction.

### 2.2. NMR Analysis 

**Figure 2:**
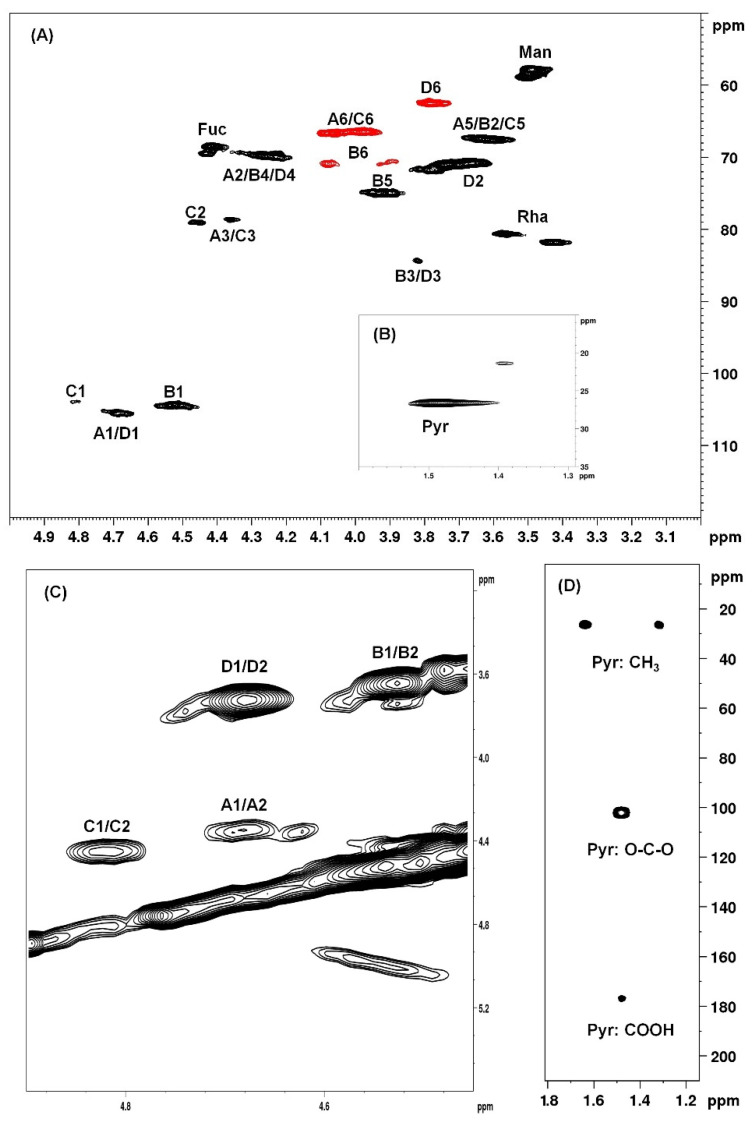
2D-NMR spectra of SP1. (**A**) ^1^H/^13^C correlation 2D-NMR spectrum (HSQCed). (**B**) ^1^H/^13^C correlation 2D-NMR spectrum (HSQCed) of pyruvate. (**C**) ^1^H/^1^H correlation 2D-NMR spectrum (COSY). (**D**) ^1^H/^13^C correlation 2D-NMR spectrum (HMBC) of pyruvate.

NMR spectroscopy analysis is the most frequently used analytical technique for the structural characterization of sulfated glycans [25]. The analysis of 1H-NMR, as seen in Figures S2 and S3, COSY, as seen in Figures S4 and S5, and HSQCed, as seen in Figure 2 and Figure S6, spectra resulted in the identification of four main structural units of SP1 and SP2, labeled A to D, as seen in Figure 2A,C. The signals of the anomeric hydrogens of units A, B, C, and D were 4.69, 4.52, 4.82, and 4.69 ppm, respectively. These anomeric values indicated that all identified units are in the β configuration [26,27]. With the chemical shifts of the anomeric hydrogens, it was possible to determine the ^1^H/^13^C correlation in an HSQCed experiment, as seen in Figure 2A.

Table 2 presents the results of the HSQCed analysis. As can be observed, SP1 and SP2 are very similar because these polysaccharides have many common structural characteristics, which can be confirmed by analyzing the spectra present in Supplementary Figures S2–S6. Therefore, only the spectra of SP1 are presented in Figure 2. The main difference between the two SPs was the signal of position 4 of unit B: in SP1, it was at 4.27/69.8 ppm, whereas in SP2, it was at 5.00/78.3 ppm. This result indicated the presence of a sulfate group at position 4 of a galactose in SP2 [28]. Furthermore, anomeric signals of 4.90/103.6 and 4.87/104.5 ppm were identified in SP2, but it was not possible to identify the other positions of the unit. These values have been described as characteristic of pyruvate galactan structural units in positions 4 and 6 [29] and in positions 3 and 4 [30], respectively.

Through the analysis of HSQCed spectra, it was also possible to determine each of the 4 structural unit constituents of the SPs. Unit A presents anomeric correlation between hydrogen and carbon at 4.69/105.5 ppm. In addition, it was possible to observe the presence of a signal at 1.48/26.4 ppm corresponding to pyruvate, as seen in Figure 2B, as one of the substituents of units A and C, as seen in Table 2, linked at O-4 and O-6. The presence of pyruvate was also confirmed in the HMBC spectrum, as seen in Figure 2D. Thus, both the A and C units are in the form of 4,6-*O*-(1-carboxyethylidene)-β-d-galactose or →3)4,6Pyr-β-d-Gal*p*-(1→. Unit B has 4.52/104.4 ppm as H1/C1 correlation. In addition, there are chemical shifts at 3.81/84.4 and 4.00/71.0 ppm at positions 3 and 6, respectively, which are characteristics of β(1→3,6)-linked units [28]. Unit C presents an anomeric signal at 4.82/103.9 ppm, but also shows signals of pyruvylation at positions 4 and 6. However, when compared to unit A, it is possible to observe that positions C1 and C2 are deshielded, probably due the presence of a sulfate group at C2 [29]; this unit was identified as →3)4,6Pyr-β-D-Gal*p*2S-(1→. Unit D has 4.69/105.5 ppm as H1/C1 correlation and a chemical shift of position 3 (3.81/84.4 ppm) corresponding to →3)-β-D-Gal*p*-(1→ [28].

We detected some signals that are not part of any of the four systems we have marked, as seen in Table 2. Costa et al. [23] showed CBB-F1.0 made of galactose:xylose:mannose (1.0:0.6:0.1) and traces of fucose and rhamnose. Thus, these signals may be indicative of the presence of these monosaccharides in SP1 and SP2. The signal at 4.41/68.5–69.5 ppm corresponds to H5/C5 correlation of fucose units [31]. The presence of O-methyl-mannose was confirmed by the signal at 3.49/57.8–58.8 ppm [32]. We also found two signals that correspond to O-methyl-rhamnose (3.57/80.6 ppm) and (3.42/81.8 ppm) [33,34]. However, we were not able to identify spin systems that confirm the presence of these monosaccharides. Most likely, these sugars can be randomly distributed throughout the galactan structures.

**Figure 3:**
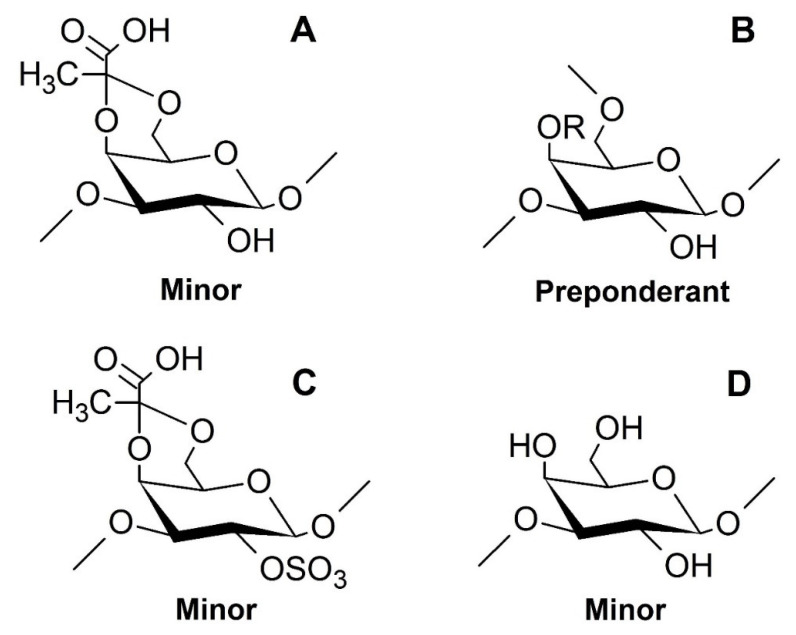
Proposed structures of the components found in the SP1 and SP2. The radical R corresponds to hydrogen atoms (H) or sulfate groups (SO_4_); however, for SP1, R = H. (**A**): →3)4,6Pyr-β-d-Gal*p*-(1→; (**B**): →3,6)-β-d-Gal*p*-(1→, for SP1 and →3,6)-β-d-Gal*p*4S-(1→, for SP2; (**C**): →3)4,6Pyr-β-d-Gal*p*2S-(1→; (**D**): →3)-β-d-Gal*p*-(1→.

Overall, the studies of NMR spectra ensure that the →3,6)-β-d-Gal*p*-(1→ is the predominant unit of these polysaccharides, as seen in Figure 3B. However, galactopyranosyl units linked by β1→3 linkages are also found, as seen in Figure 3D, as there are units containing 3,4-*O*-(1’ carboxy)-ethylidene, as seen in Figure 3A, and 2-*O*-sulfate, as seen in Figure 3C.

The presence of β-d-galactans in green algae has also been reported in other works. Ciancia et al. [35] identified a 3-linked β-d-galactan, partially sulfated at C6 and pyruvylated at positions 4 and 6, from *Bryopsis plumosa*. Species of the genus *Codium* synthesize galactans consisting of partially sulfated C4 and/or C6 β-d-galactopyranose residues with significant pyruvate content [26,27,28,30]. Sulfated and pyruvylated galactans were also isolated from the tropical green algae species *Penicillus capitatus*, *Udotea flabellum*, and *Halimeda opuntia* [29]. However, studies on sulfated and pyruvylated galactans obtained from green algae with immunostimulating properties are scarce. Because of that, a question arose. Would these galactans be responsible for the immunomodulatory activity that was observed in CCB-1.0 and was it shown by Barbosa et al. [24]? Therefore, the effect of these galactans on the levels of some immunomodulation mediators (NO, ROS, and cytokines) was evaluated.

### 2.3. Cell Viability

**Figure 4:**
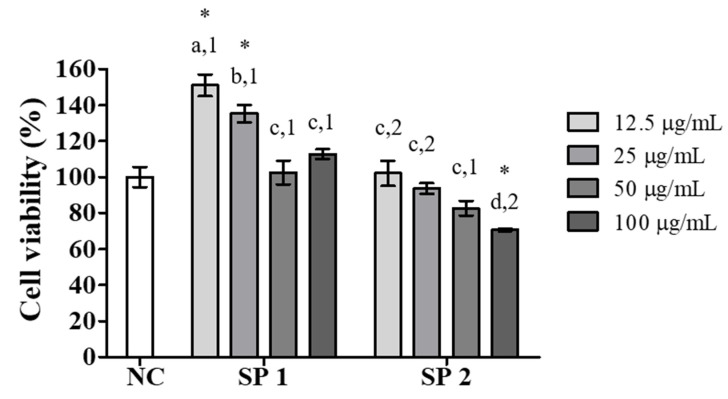
Effects of SP1 and SP2 of *C. cupressoides* on the cell viability of RAW 264.7 macrophages. NC—negative control. The data are presented as the mean ± standard deviation (*n* = 3). Different letters represent statistically significant differences between the different SPs (*p* < 0.05). Different numbers represent statistically significant differences between the same concentrations of the two SPs (*p* < 0.05). * represents samples that presented statistically significant differences in relation to the negative control (*p* < 0.05).

Macrophages play several roles in the biology of organisms, including development, homeostasis, and repair, as well as the immune response against pathogens [36]. The evaluation of the viability of RAW 264.7 murine macrophages has previously been used as an indicator of the activation of macrophages and, subsequently, for the characterization of the immunomodulatory potential of new compounds with biomedical applications [37]. Among these analyses, the capacity to reduce MTT to formazan crystals by mitochondrial dehydrogenases is an important measure for evaluating cell viability. Therefore, the effect of SP1 and SP2 on the ability of RAW 264.7 macrophages to reduce MTT after an exposure period of 24 h at concentrations ranging from 12.5 to 100 μg/mL was initially evaluated. As shown in Figure 4, a statistically significant (*p* < 0.05) increase in MTT reduction capacity was promoted by SP1 at concentrations of 12.5 and 25 μg/mL, whereas for the other concentrations, the values did not differ from that of the negative control. For SP2, the cell viability did not differ from that of the negative control, except at the highest concentration (100 μg/mL), which caused a statistically significant decrease (*p* < 0.05) in MTT reduction capacity of around 30%. In this case, the higher SP2 concentration may have interfered with mitochondrial metabolism and consequently led to a reduction of cell viability. These results suggest that at concentrations less than 100 μg/mL, SP1 and SP2 do not cause considerable cytotoxic effects on RAW 264.7 macrophages. For the following analyses, the concentration of 100 μg/mL of SP2 was excluded because it promoted a reduction in cell viability and potentially had a cytotoxic effect.

### 2.4. NO Production

**Figure 5:**
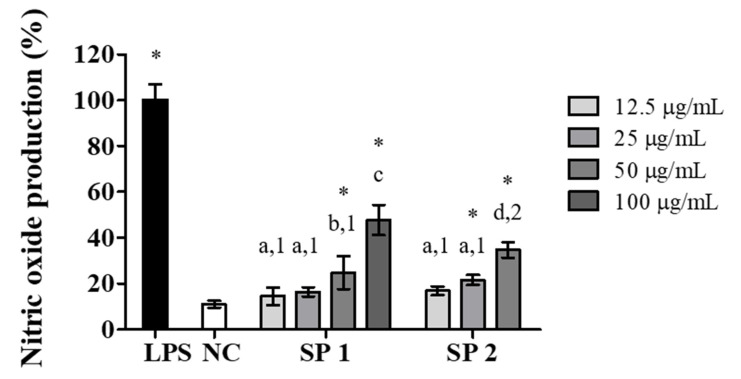
Effects of SP1 and SP2 of *C. cupressoides* on NO production. NC—negative control; LPS—bacterial lipopolysaccharide (2 µg/mL). The data demonstrate the mean ± standard deviation (*n* = 3). Different letters represent statistically significant differences between SPs concentrations (*p* < 0.05). Different numbers represent statistically significant differences between the same concentrations of different SPs (*p* < 0.05). * represents samples that had a statistically significant difference in relation to the negative control (*p* < 0.05).

Stimulatory effects of algae polysaccharides on macrophages result in the production of NO through the induction of the enzyme inducible nitric oxide synthase [38,39]. NO is a highly reactive molecule that is important for the functioning of the immune system, and it has cytotoxic effects on pathogenic microorganisms and cancer cells [40]. Therefore, NO production in the supernatant of RAW 264.7 murine macrophages stimulated with *C. cupressoides* SPs was evaluated. The NO production induced by SP1 and SP2 at concentrations between 12.5 and 100 μg/mL, expressed in relation to the amount of NO produced in the positive control (2 μg/mL LPS), is shown in Figure 5. SP1 promoted a statistically significant increase (*p* < 0.05) in the production of NO at concentrations of 50 and 100 μg/mL, whereas for SP2, the concentrations of 25 and 50 μg/mL showed the greatest stimulatory effect on the production of NO. These results were similar to those reported in other works that evaluated the effects of SPs obtained from different species of seaweed on the ability of induce NO production in macrophages [41,42,43].

Studies on the immunostimulatory effect of polysaccharides have revealed that these molecules are capable of binding to several receptors on the surface of macrophages, which can activate several intracellular signaling pathways. As a result of this activation, the expression of genes that encode different inflammatory mediators, such as NO and cytokines, is initiated [44,45,46]. Some structural requirements, necessary for the immunostimulatory activity of algae polysaccharides, have been identified by Leiro et al. [47]. They found that the desulfation of polysaccharides from *Ulva rigida* was a determining factor for its immunostimulatory activity. The presence of the sulfate group was also shown to be an important characteristic for the effect of *Sargassum angustifolium* polysaccharides on NO production [48]. For SPs from *Ulva intestinales*, the lower molecular weight of one of the fractions was found to be a fundamental characteristic for immunostimulatory activity [13]. In another study, Bahramzadeh et al. [49] proposed that the compaction of the SPs obtained from *Cystoseira indica* seemed to be a more determinant characteristic for its immunostimulatory capacity than the size of the molecule and its degree of sulfation. However, the monosaccharide composition, sulfate content, and ultrastructure of *C. lentifera* polysaccharides appeared to have been crucial for their effect on NO production in RAW 264.7 macrophages [18]. Therefore, a systematic and in-depth study that aimed to relate the individual and/or combined structural characteristics of *C. cupressoides* SPs and their immunostimulatory effect was needed.

### 2.5. Production of Intracellular ROS

**Figure 6:**
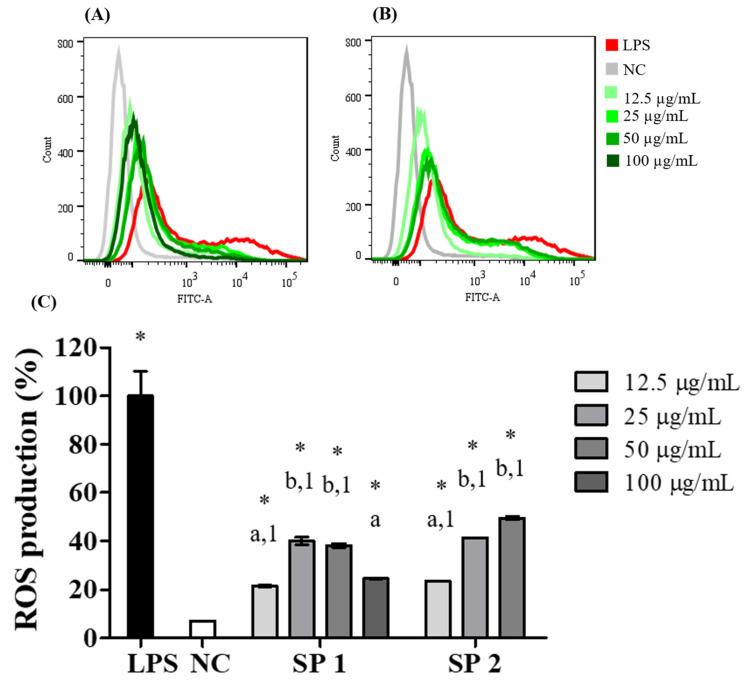
Production of ROS. Histograms representative of the effects of different concentrations of SP1 (**A**) and SP2 (**B**) on intracellular ROS production quantified by flow cytometry. NC—negative control; LPS—bacterial lipopolysaccharide (2 µg/mL). (**C**) Percentage of intracellular ROS production in relation to that of LPS-stimulated cells. The data are presented as the mean ± standard deviation (*n* = 3). Different letters represent statistically significant differences between SPs concentrations (*p* < 0.01). Different numbers represent statistically significant differences between the same concentrations of different SPs (*p* < 0.01). * represents samples that had a statistically significant differences in relation to the negative control (*p* < 0.01).

During the processes of the recognition and response to harmful cell agents, phagocytic cells produce NO and ROS [50]. Thus, ROS production is an important indicator of macrophage function [51]. Because the treatment of RAW 264.7 macrophages with the purified SPs of *C. cupressoides* promoted a stimulatory effect on NO production, it was determined whether there would be a similar effect on the production of intracellular ROS. Figure 6 shows the effect of treatment with SP1 and SP2 on the production of ROS after a 24 h exposure period at concentrations ranging from 12.5 to 100 μg/mL. The displacements of the histograms shown in A and B indicated that the two SPs promoted an increase in ROS production. Analysis of the fluorescence intensity emitted by cells treated with the SPs in relation to that of the positive control (2 μg/mL LPS) revealed a statistically significant increase (*p* < 0.01) in all evaluated conditions, as seen in Figure 6C. These results are consistent with the effects of immunostimulating polysaccharides on ROS production reported in other studies. Wang et al. [52] reported that SPs from *Ascophyllum nodosum* significantly increased intracellular ROS levels in macrophages. In another study, sulfated galactans from the algae *Gracilaria lemaneiformis* also promoted stimulatory effects on ROS production [53], whereas the treatment of peritoneal macrophages with arabinogalactan and fucoidan increased the production of reactive oxygen and nitrogen species [54]. Although the increase in ROS production, induced by SP1 and SP2, was lower than that observed in the positive control, their stimulatory effects can be considered to be relevant and safe for macrophage activation because the excess production of ROS can lead to severe oxidative damage in cells.

### 2.6. Proinflammatory Cytokine Production

**Figure 7:**
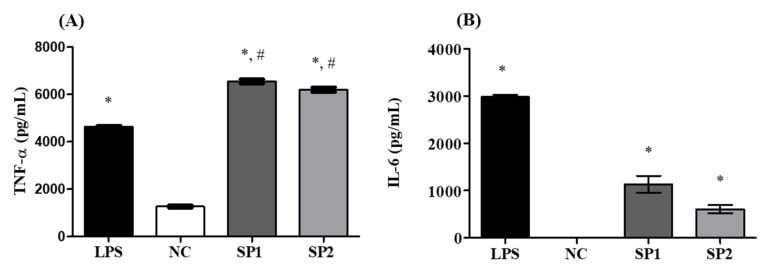
Production of the proinflammatory cytokines TNF-α (**A**) and IL-6 (**B**). NC—negative control; LPS—bacterial lipopolysaccharide (2 µg/mL). The data are presented as the mean ± standard deviation. * represents samples that had a statistically significant differences in relation to the negative control (*p* < 0.01). # represents statistically significant increases (*p* < 0.01) in relation to LPS. SP1 and SP2 were evaluated at concentrations of 100 μg/mL and 50 μg/mL, respectively. IL-6 was not detected in the NC group.

Secretion of proinflammatory cytokines by activated macrophages is directly involved in the defense against pathogen invasion. These molecules play an important regulatory role in cell growth, proliferation, and immunity [55]. When exposed to immunostimulatory agents, macrophages secrete inflammatory mediators, including NO, ROS, and cytokines such as TNF-α and IL-6 [56,57]. Here, both cytokines were quantified in the supernatant of RAW 264.7 macrophages exposed to SP1 and SP2 at concentrations of 100 and 50 μg/mL, respectively. As shown in Figure 7A, a statistically significant increase (*p* < 0.01) of TNF-α production was observed in cells treated with SP1 and SP2, compared to that in the negative control. It should be noted that the effect of these SPs on the production of TNF-α was higher than that in the cells stimulated with LPS (positive control). SP1 and SP2 also promoted a statistically significant increase (*p* < 0.01) in the levels of IL-6, compared to that in the negative control, as seen in Figure 7B.

The quantification of the proinflammatory cytokines TNF-α and IL-6 in macrophage cultures is an important assessment in studies on the immunomodulatory effects of polysaccharides obtained from algae. SPs from *Cyclocarya paliurus* increased the immunostimulatory activity of RAW macrophages through the production and secretion of various inflammatory mediators, including TNF-α and IL-6 [58]. In another study, the fucoidan from *Undaria pinnatifida* had a potent stimulatory effect on the production of TNF-α and IL-6 [59]. Similar effects on TNF-α and IL-6 secretion in RAW 264.7 macrophages were also observed in the study by Liu et al. [43] that evaluated the effects of *Porphyra haitanensis* SPs as well as in the work by Ren et al. [53] that evaluated the immunostimulatory effect of the polysaccharides from *Gracilaria lemaneiformis*. In a previous study, we showed that CCB-F1.0 promoted a significant increase in the production of IL-6 and TNF-α [24]. However, to investigate this effect, CCB-F1.0 was tested in concentrations 8 and 16 times higher than SP1 and SP2, respectively. These findings indicate that the purification process enabled isolation of the compounds with more proinflammatory effect. Therefore, *C. cupressoides* SPs may play an important role in stimulating the immune response mediated by macrophages.

## 3. Materials and Methods 

### 3.1. Materials

1,9-Dimethyl-methylene blue zinc chloride double salt (DMMB), 2′,7′-dichlorofluorescin diacetate (DCFH-DA), 3-(4,5-dimethylthiazolyl-2)-2,5-diphenyltetrazolium bromide (MTT), deuterium oxide (D_2_O), and Griess reagent were purchased from Sigma Chemical Company (St. Louis, MO, USA). Dulbecco’s modified Eagle’s medium (DMEM) and fetal bovine serum (FBS) were obtained from Cultilab (Campinas, SP, Brazil). Penicillin and streptomycin were purchased from Gibco (Fort Worth, TX, USA). The kit for cytokine analysis was purchased from BD Biosciences (San Jose, CA, USA). Lipopolysaccharide (LPS), *Escherichia coli* 055: B5 was obtained from Santa Cruz Biotechnology (Dallas, TX, USA). All the other solvents and chemical products were of analytical grade.

### 3.2. Seaweed Collection

The green macroalgae *C. cupressoides* var. *flabellata* was collected in the city of Nísia Floresta, on the southern coast of the state of Rio Grande do Norte, Brazil. After the collection, the seaweed was transported to the Laboratório de Biotecnologia de Polímeros Naturais of the Biochemistry Department, Universidade Federal do Rio Grande do Norte, for the removal of epiphytic species, sediment, and encrusted organisms and subsequent extraction of their SPs. The material collection occurred under authorization of Brazilian National Management System Genetic Heritage and Associated Traditional Knowledge (loose translation) SisGen n° A0D4240.2.1.1. 

### 3.3. Extraction of SPs

To obtain the SPs-rich extract, the seaweeds were dried, crushed, delipidated with ethanol, and submitted to proteolysis. The proteolytic digestion was conducted with 100 g of this powdered seaweed, 0.25 M NaCl (500 mL) pH = 8.0, and a mixture of alkaline proteases (Prolav 750, Prozyn Biosolutions, São Paulo, SP, Brazil) at 15 mg/g powdered seaweed; for 18 h at 60 °C. The SPs-rich extract was obtained after filtration and centrifugation (10,000× *g* for 20 min, 4 °C), and it was submitted to fractionation step as described by Costa et al. [23]. Briefly, 0.3 volumes of propanone (4 °C) was dropped to the SPs-rich extract under gentle agitation and maintained for 24 h. The material was centrifuged (10,000× *g* for 20 min, 4 °C), dried, and kept in the dark until further use. The fractionation was repeated by adding 0.5, 1.0, and 2.0 volumes of propanone to the supernatant. Based on the propanone volumes used, the fractions were named CCB-F0.3, CCB-F0.5, CCB-F1.0, and CCB-F2.0, respectively.

### 3.4. Purification of SPs by Liquid Ion Exchange Chromatography

The SPs of the CCB-F1.0 fraction were purified with a fast protein liquid chromatography (FPLC) system at ÄKTA-GE (Healthcare Life Sciences, Little Chalfont, Bucks, UK) using a 5 mL HiTrap DEAE FF column (GE Healthcare, Westborough, MA, USA). To prepare the sample, the CCB-F1.0 fraction was solubilized in 0.25 M NaCl at concentration of 5 mg/mL, filtered using 0.22 μm filters, and injected into the column (1 mL). The SPs bound to the column were eluted by applying a stepwise NaCl gradient (0.525, 0.675, and 0.9 M), based on a first elution with continuous gradient ranging from NaCl 0.25 to 3.0 M. All peaks were monitored by DMMB metachromasia and absorbance at 525 nm [60]. This procedure was repeated ten times. Then, the material (each peak) obtained was pooled and dialyzed (MW cut-off of 6 kDa) against distilled water and lyophilized.

### 3.5. Physicochemical Analysis

Total sugar content determination was performed by the phenol-sulphuric acid method using galactose as the reference sugar [61]. Sulfate content was determined by the barium chloride-gelatin method [62]. Protein content was measured as described by Bradford [63]. Monosaccharide composition was determined by nuclear magnetic resonance spectroscopy as described by Sassaki and coworkers (2014) [64]. Briefly, samples (5 mg) were hydrolyzed with 2 M HCl for 4 h at 100 °C. Then, the solution was neutralized, evaporated, and the residue dissolved in D_2_O for NMR analysis (~600 μL). A diluted D_2_O–H_2_SO_4_ solution was used for adjusting pH value to 3.5. Finally, quantitative-HSQC (Q-HSQC) spectra were obtained at 27 °C using a hsqcetgpsisp2.2 pulse sequence on Bruker spectrometers (Bruker, Billerica, MA, USA).

### 3.6. Molecular Weight and Homogeneity Determination

Molecular weight and homogeneity SPs from *C. cupressoides* were determined by gel permeation chromatography (GPC). Each fraction was dissolved to a final concentration of 10 mg/mL and applied to a column containing Sephadex^®^ G-100 (135 × 1 cm i.d.; Sigma Chemical Company, St. Louis, MO, USA). GPC was performed using an isocratic elution mode. The molecular weight was estimated by reference to a calibration curve made by dextran standards (10, 40, 70, 147, and 500 kDa). Homogeneity of SPs was evaluated by chromatographic profile. The SPs elution was monitored by sugar determination according to Dubois et al. [61].

### 3.7. NMR Spectroscopy

NMR spectra were obtained using a Bruker Avance III Ascend 600 MHz (14.1 T) spectrometer (Billerica, MA, USA) equipped with a 5 mm inverse probe. Each sample had its ^1^H 90° pulse calibrated and transmitter frequency offset calculated = 1881.69 Hz (SP1) and 2822.59 Hz (SP2), and the value was used to all following experiments, which were conducted at 70 °C with samples (20 mg/mL) previous solubilized with deuterium oxide (D_2_O) without spinning. 1D-NMR spectra were obtained using a presat sequence pulse (zgpr) to suppress solvent peak. Presat experiments were performed with a spectral width (SWH) of 4795.396 Hz, size of fid (TD) = 64k, and number of scans (NS) = 8. 2D-NMR homonuclear (^1^H–^1^H) COSY (Correlation Spectroscopy) analysis were performed using Bruker’s cosygpprqf pulse sequence with SWH = 4795.396 Hz, TD = 2048 (F2) × 256 (F1), number of dummy scans (DS) = 16, NS = 16, and relaxation delay = 1.5 s. 2D-NMR heteronuclear (^1^H–^13^C) HSQCed (Edited Heteronuclear Single Quantum Coherence) analysis were performed using Bruker’s hsqcedetgpsisp2.2 pulse sequence with SWH = 4795.396 Hz (F2) × 16667.754 Hz (F1), TD = 2048 (F2) × 256 (F1), DS = 16, NS = 32, and relaxation delay = 1.0 s. HMBC (Heteronuclear Multiple Bond Correlation) analysis were performed using Bruker’s hmbcgpndqf pulse sequence with SWH = 4789.272 Hz (F2) × 20124.867 Hz (F1), TD = 2048 (F2) × 256 (F1), DS = 16, NS = 46, and relaxation delay = 1.0 s. The chemical shift of ^1^H and ^13^C were expressed in δ (ppm) relative to TMSP (trimethylilsilylpropionate) as an internal standard (δ = 0 ppm). NMR spectra analysis was conducted using Bruker’s TopSpin v 4.0.6 software (Bruker, Billerica, MA, USA). The proposed structures were designed using ChemSketch v.12.0 software (ACD/Labs, Toronto, ON, Canada).

### 3.8. Cell Culture

The RAW 264.7 macrophage cell line (ATCC number TIB-71) was cultured in DMEM supplemented with FBS (10% v/v) and antibiotics (100 U/mL penicillin and 100 μg/mL streptomycin). The cells were incubated at 37 °C in a humidified atmosphere with 5% CO_2_. For maintenance of the cells, the culture medium was changed every three days, and the cells were further subcultured at the 80% confluency using a cell scraper.

### 3.9. MTT Reduction Test

The ability of RAW 264.7 macrophages to reduce MTT was evaluated according to the previously described method of Mosmann [65] to analyze the effect of SPs on cell viability. Initially, the cells were seeded in 96-well plates at a density of 1 × 10^4^ /well. After treatment with the SPs at the different concentrations tested (12.5–100 μg/mL) for 24 h, the culture medium was replaced with 100 μL of MTT (1 mg/mL dissolved in DMEM). Then, the cells were incubated for 4 h at 37 °C. Subsequently, the culture supernatant was discarded, and the crystals of formazan were solubilized in ethanol, 100 μL/well. Absorbance was measured with an Epoch microplate spectrophotometer (Biotek Instruments Inc., Winooski, VT, USA) at 570 nm. Cell viability was calculated in relation to the negative control using the formula: % viability = (Atest/AControl) × 100, in which Atest corresponds to the absorbance of the experimental group and Acontrol corresponds to the absorbance of the negative control.

### 3.10. NO Production

The nitrite levels released in the supernatant of the cultured RAW 264.7 cells were quantified to evaluate the immunostimulatory effect of the SPs of *C. cupressoides*, as described previously [66]. Initially, the cells were cultured (3 × 10^5^/well) in 24-well plates and exposed to different concentrations of the purified SPs (12.5–100 μg/mL) for 24 h. LPS (2 μg/mL) was used as a positive control. After the treatment time, 100 μL of the supernatant was collected and mixed with 100 μL of Griess reagent and incubated for 10 min at room temperature in the dark. Absorbance was measured at 540 nm with a microplate spectrophotometer. Sodium nitrite was used as a standard, and the results were expressed as the percentage of nitrite production in relation to that of the positive control (LPS) according to the formula: % of nitrite production = (Atest/ALPS) × 100, in which Atest corresponds to absorbance of the experimental group and ALPS corresponds to the absorbance of LPS (positive control).

### 3.11. Intracellular ROS Production

The levels of intracellular oxygen reactive species were evaluated by quantifying the fluorescence emitted by 2′,7′-dichlorofluorescein, the oxidized form of 2′,7′-dichlorofluorescein diacetate (DCFH-DA). For this, RAW 264.7 macrophages were cultured (3 × 10^5^/well) in 24-well plates and exposed to SPs for 24 h. LPS (2 μg/mL) was used as a positive control. After treatment, the supernatant was discarded, cells were washed with phosphate buffered saline (PBS), and 100 µM DCFH-DA in DMEM containing 1% FBS was added, followed by incubation at 37 °C for 2 h. Then, the DCFH was removed, the cells were washed twice with PBS, and the emitted fluorescence was measured on a flow cytometer (FACSCanto II, BD Biosciences, Eugene, OR, USA) with FACSDiva software, version 6.1.2 (Becton Dickson, Franklin Lakes, NJ, USA). The results were analyzed in FlowJo software (FlowJo, Ashland, OR, USA) and expressed as % of fluorescence emitted relative to that of LPS-treated cells.

### 3.12. Cytokine Production

For quantification of the cytokines IL-6 and TNF-α, the supernatant of the cells that were exposed to SPs in the experiment to evaluate ROS levels was used. The cytokines were quantified using the BD Cytometric Bead Array (CBA) Mouse Th1/Th2/Th17 Cytokine Kit according to the manufacturer’s instructions using a flow cytometer (FACSCanto II, BD Biosciences, OR, USA). The results were recorded with FACSDiva software and analyzed using FCAP Array software, version 3.0 (BD, Franklin Lakes, NJ, USA).

### 3.13. Statistical Analysis

Statistical analysis was performed using Prism 5 (GraphPad Prism, version 5.00, San Diego, CA, USA). Results were expressed as the mean ± standard deviation. Statistical differences between the groups were assessed using analysis of variance and the Student Newman–Keuls post-test. Differences were considered significant at *p* < 0.05.

## 4. Conclusions

In this study, two sulfated and pyruvated galactans were obtained from an SP-rich fraction from *C. cupressoides* seaweed. Structurally, both galactans are similar, differing only by the presence of a sulfation at position 4 in one of the SP2 units. In addition, the galactans had an immunostimulatory effect, as evidenced by the increased production of NO, ERO, and the cytokines IL-6 and TNF-α. These findings indicate that sulfation at C-4 is not essential for the immunomodulatory action of these galactans. Overall, these results suggest that *C. cupressoides* SPs represent potential candidates for the development of new products with immunomodulatory properties of biomedical interest, with applications including the treatment of hypoimmunity or immunodeficiency conditions.

## Figures and Tables

**Table 1:** Chemical composition of CCB-F1.0 and purified sulfated polysaccharides from *Caulerpa cupressoides*.

Samples	Sulfate (%)	Protein (%)	Molar Ratio			
			Gal	Man	Xyl	SO_4_
CCB-F1.0	17.8 ± 0.2	0.19 ± 0.03	1	0.1	0.6	0.56
SP1	9.35 ± 0.17	0.25 ± 0.04	1	0.08	n.d.	0.27
SP2	12.4 ± 0.1	0.20 ± 0.03	1	0.09	n.d.	0.98

Gal = galactose; Man = mannose; Xyl = xylose, SO_4_ = sulfate, n.d. = not detected.

**Table 2:** Signal assignments (ppm) of NMR spectra of SP1 and SP2 from *C. cupressoides*.

Structural Unit	Chemical Shifts (ppm)					
	H-1/C-1	H-2/C-2	H-3/C-3	H-4/C-4	H-5/C-5	H-6/C-6
SP1						
A	4.69/105.5	4.27/69.8	4.36/78.7	3.79/71.8	3.65/67.6	3.97/66.5
B	4.52/104.4	3.65/70.8	3.81/84.4	4.27/69.8	3.92/74.9	4.00/71.0
C	4.82/103.9	4.45/79.1	4.36/78.7	n.d.	3.60/67.6	3.97/66.5
D	4.69/105.5	3.73/71.0	3.81/84.4	4.27/69.8	n.d.	3.78/62.5
SP2						
A	4.69/105.5	4.27/69.8	4.36/78.7	3.79/71.8	3.65/67.6	3.97/66.5
B	4.52/104.4	3.65/70.8	3.96/83.7	5.00/78.3	3.92/74.9	4.00/71.0
C	4.82/103.9	4.56/76.8	4.50/77.3	4.12/69.8	3.60/67.6	3.97/66.5
D	4.69/105.5	3.73/71.0	3.81/84.4	4.27/69.8	n.d.	3.78/62.5
→3)4,6Pyr-β-D-Gal*p*-(1→ ^a^	4.60/105.0	4.20/69.6	4.20/79.5	n.d./71.6	3.60/67.0	3.90/65.9
→3,6)-β-D-Gal*p*-(1→ ^b^	4.53/104.5	3.72/71.3	3.85/83.7	4.23/69.8	3.92/74.9	3.92;4.02/70.8
→3)4,6Pyr-β-D-Gal*p*2S-(1→ ^c^	4.85/103.5	4.48/76.2	4.39/77.2	4.11/71.8	3.54/66.9	3.85;3.97/65.9
→3)-β-D-Gal*p*-(1→ ^b^	4.68/105.5	3.78/71.7	3.85/83.5	4.19/69.8	3.71/76.2	3.79/62.3
→3,6)-β-D-Gal*p*4S-(1→ ^b^	4.58/104.4	3.73/72.2	4.05/80.0	4.91/79.5	4.05/74.5	n.d.

n.d. = not detected. Signals reported in literature: ^a^ Fernández et al. [30]; ^b^ Bilan et al. [28]; ^c^ Arata et al. [29].

## Supplementary Materials

The following are available online at https://www.mdpi.com/1660-3397/18/5/234/s1, Figure S1: The GPC chromatogram of SP1 and SP2 on a Sephadex G-100 column; Figure S2: ^1^H-NMR spectrum of SP1; Figure S3: ^1^H-NMR spectrum of SP2; Figure S4: ^1^H/^1^H correlation 2D-NMR spectrum (COSY) of SP1; Figure S5: ^1^H/^1^H correlation 2D-NMR spectrum (COSY) of SP2; Figure S6: ^1^H/^13^C correlation 2D-NMR spectrum (HSQCed) of SP2.
