# Supplementary material for: Immunostimulatory Effect of Sulfated Galactans from the Green Seaweed Caulerpa cupressoides var. flabellata
Source: Mar Drugs. 2020 Apr 29;18(5):234. doi: 10.3390/md18050234 (PMC7281474; doi:10.3390/md18050234)
Supplement: Supplementary file 1 [file marinedrugs-18-00234-s001.pdf]

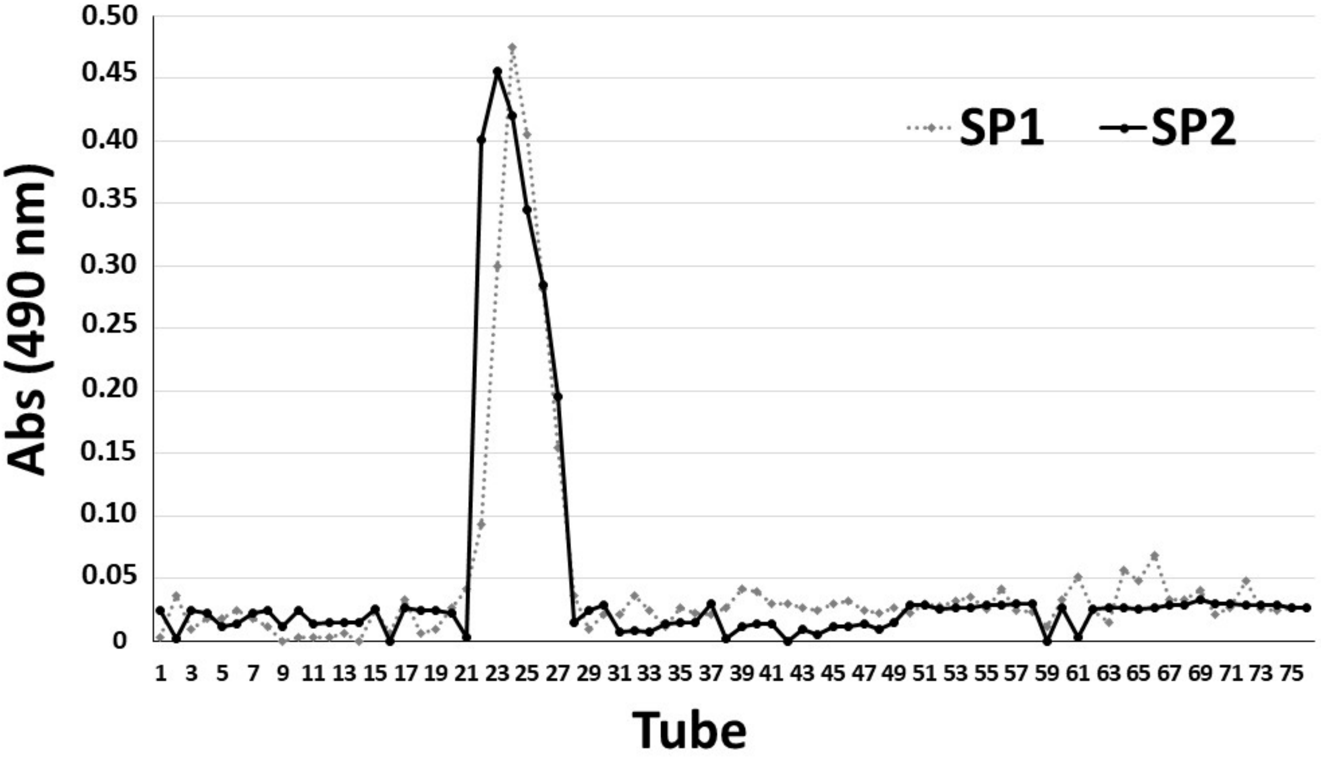

Figure S1 – The GPC chromatogram of SP1 and SP2Fon a Sephadex G-100 column

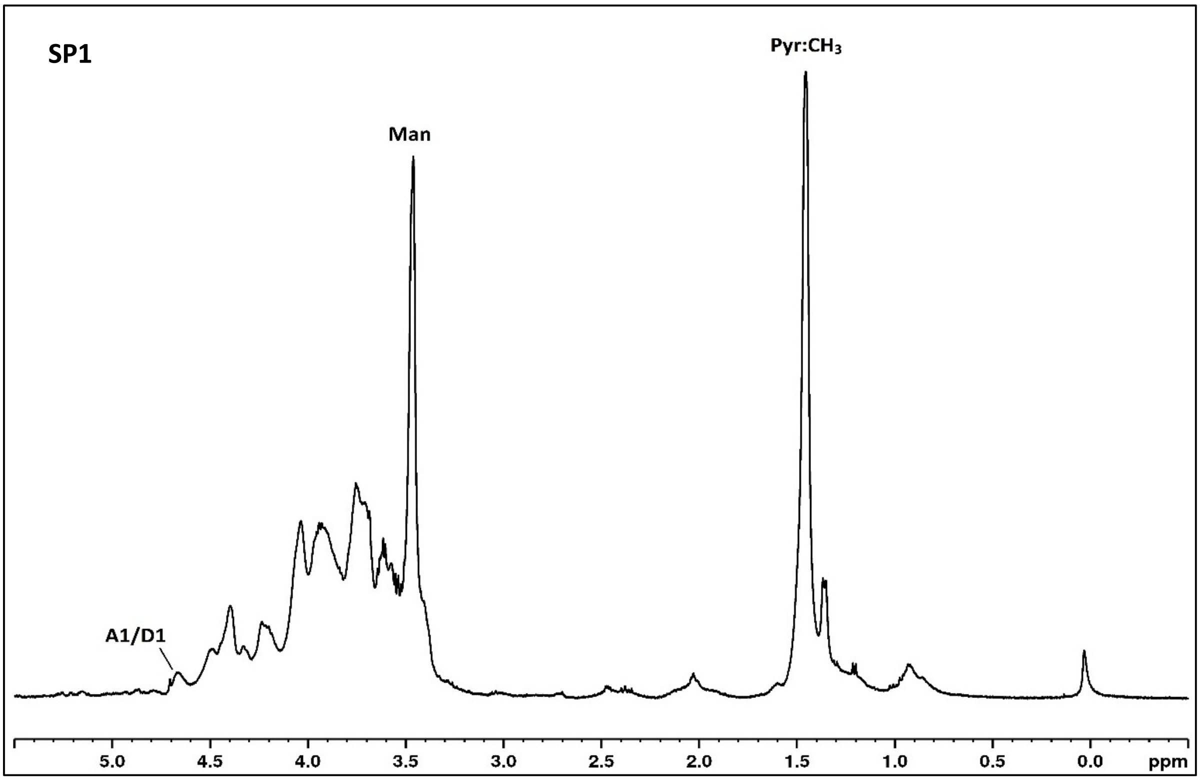

Figure S2 - ^{1}H-NMR spectrum of SP1.

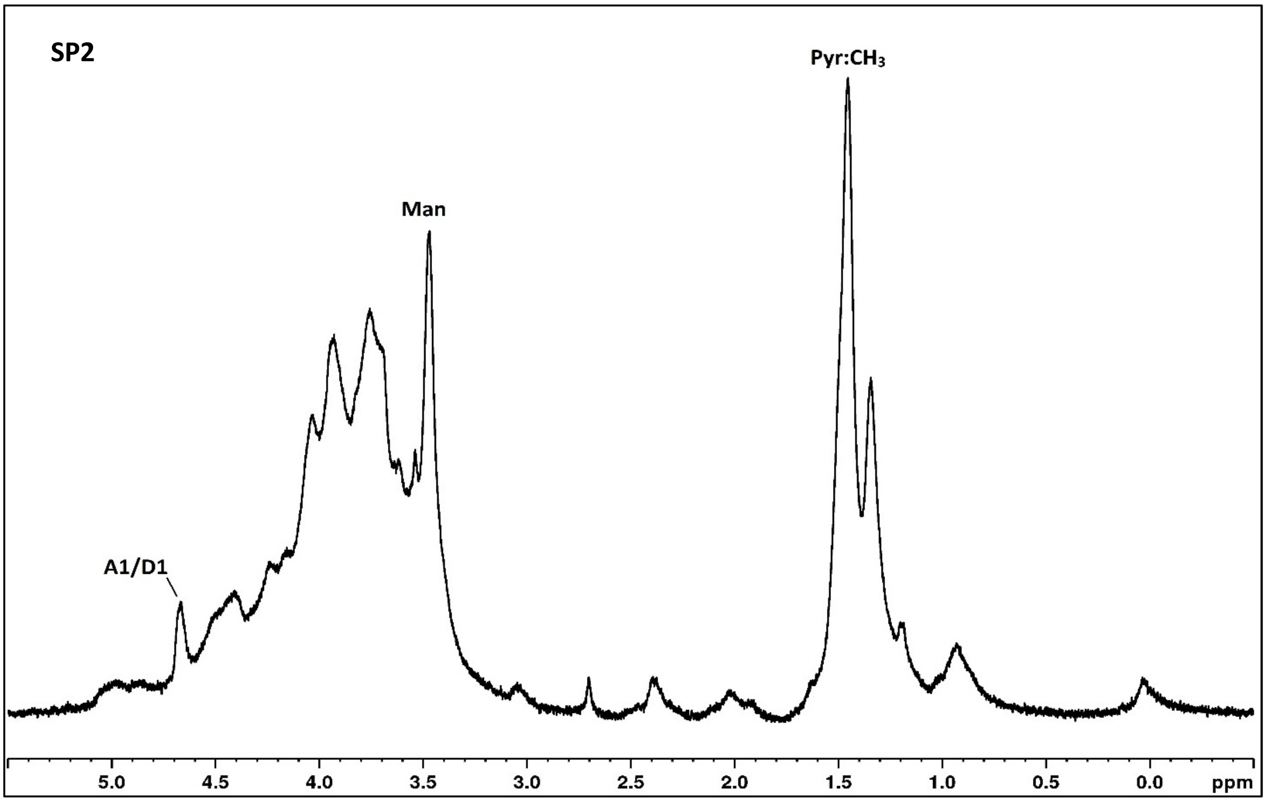

Figure S3 - ^{1}H-NMR spectrum of SP2.

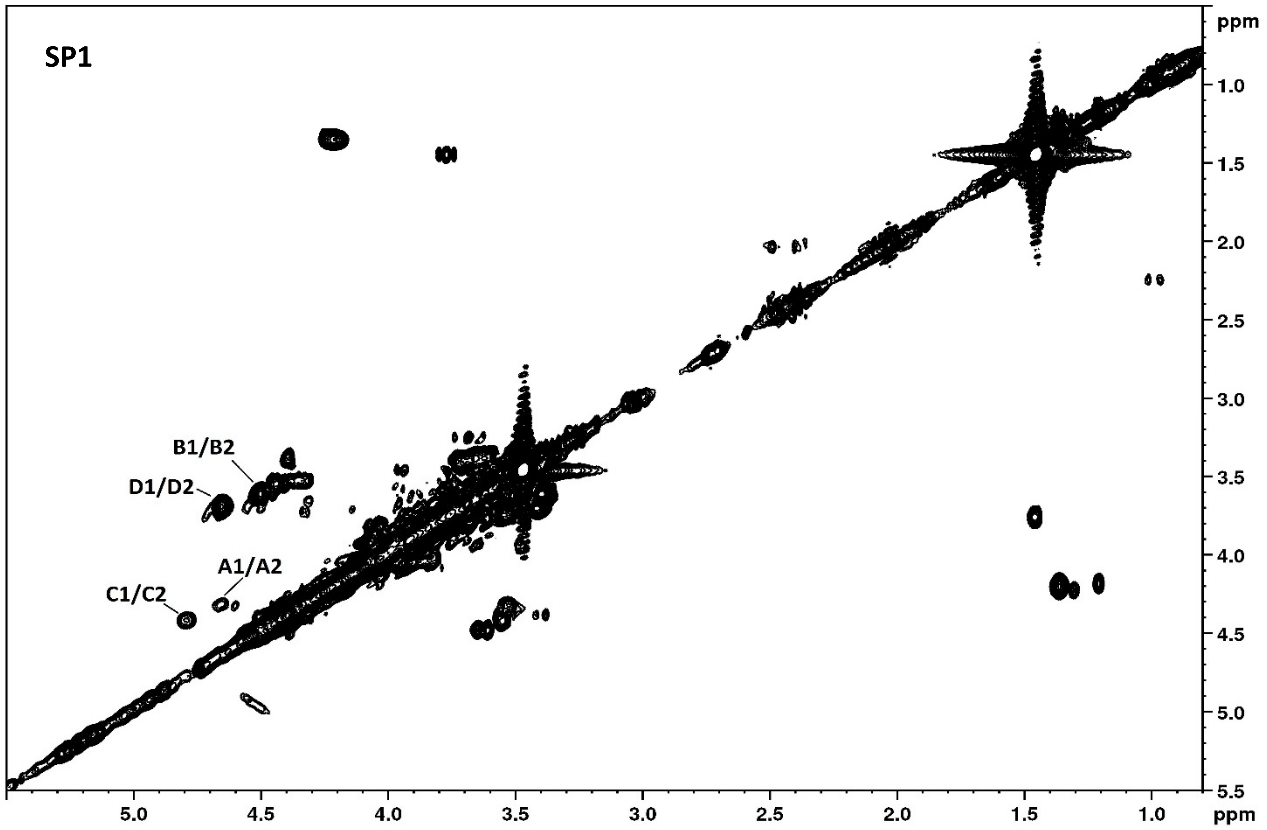

Figure S4 - $^1H/^1H$ correlation 2D-NMR spectrum (COSY) of SP1.

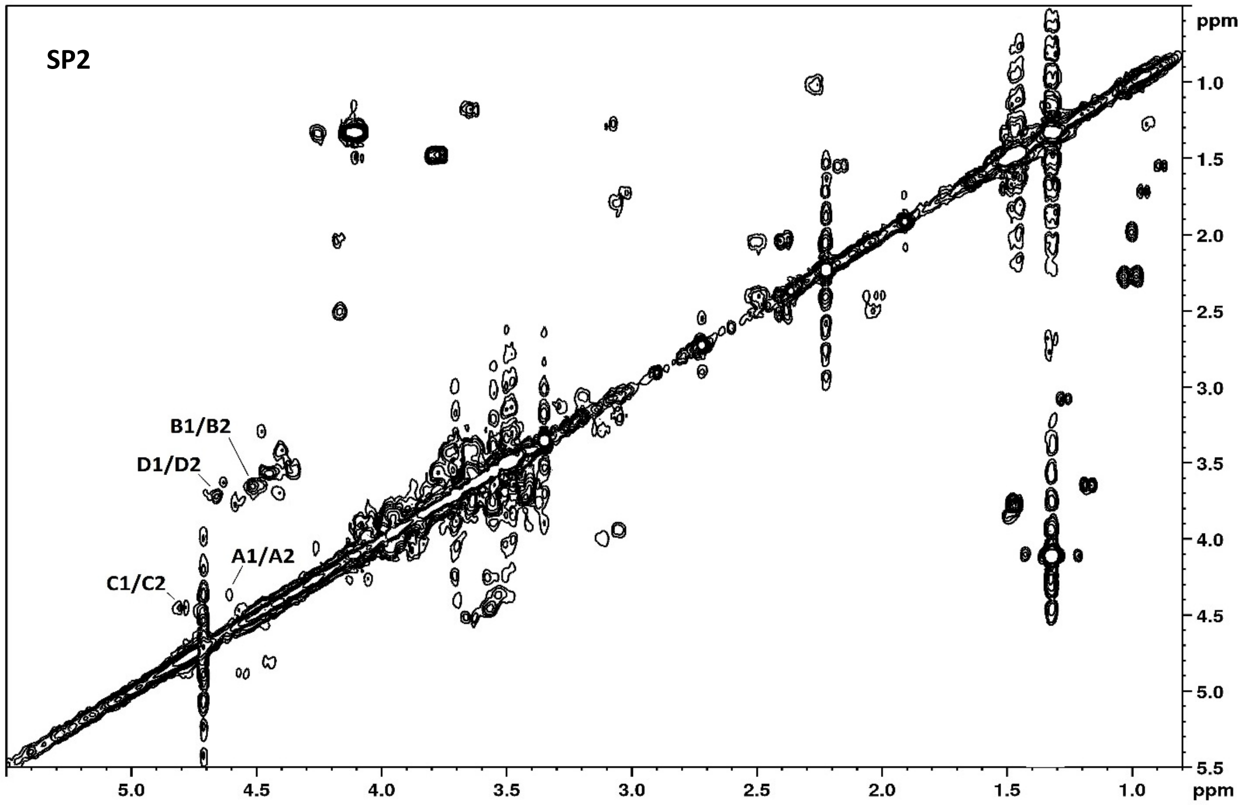

Figure S5 – $^{1}H/^{1}H$ correlation 2D-NMR spectrum (COSY) of SP2.

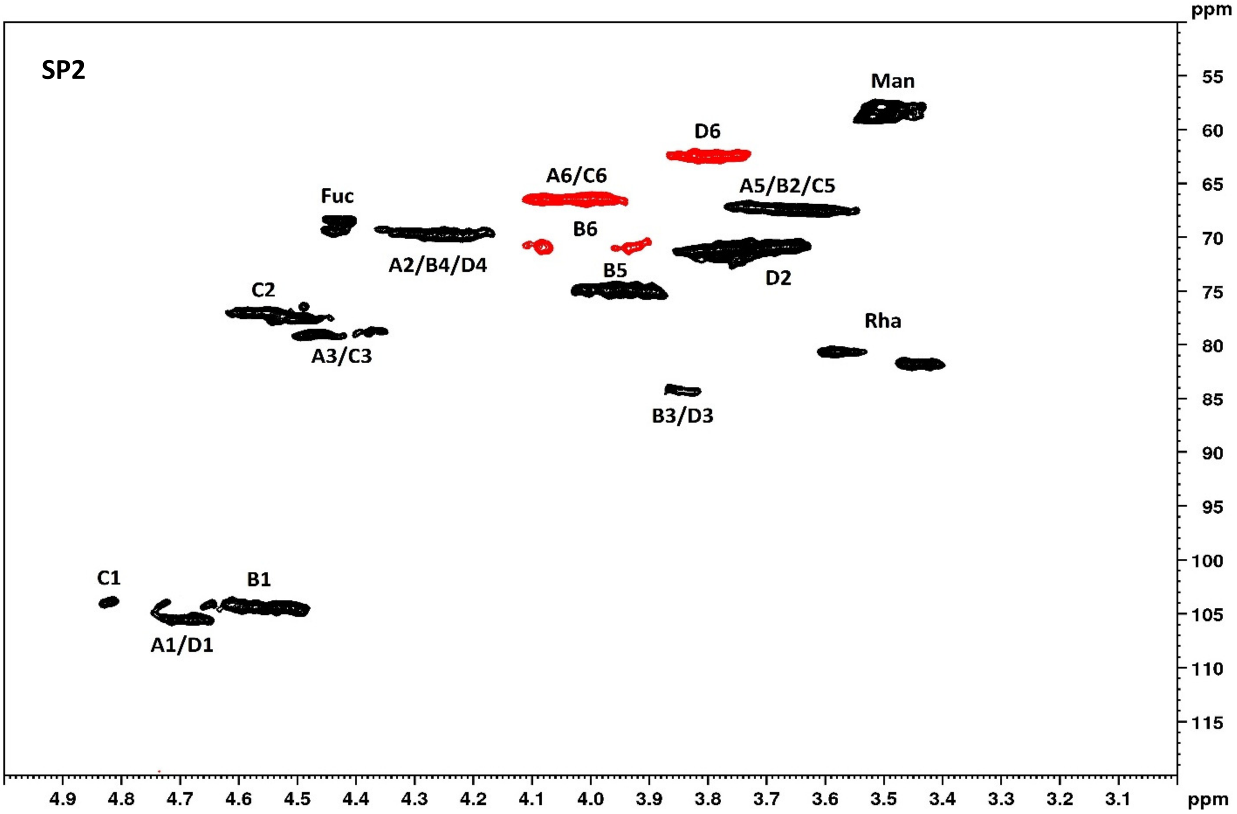

Figure S6 - $^{1}H/^{13}C$ correlation 2D-NMR spectrum (HSQCed) of SP2.
